# Diagnostic Approaches to Invasive Candidiasis: Challenges and New Perspectives

**DOI:** 10.1007/s11046-025-01035-4

**Published:** 2025-12-13

**Authors:** Hana Slepčanová, Radim Dobiáš, Andrea Beatriz Sermeño Langer, Marcela Káňová, Petr Hamal

**Affiliations:** 1 Present Address: Institute of Laboratory Medicine, University Hospital Ostrava, 17. listopadu 1790, 70852 Ostrava, Czech Republic; 2 Institute of Laboratory Medicine, Faculty of Medicine, University of Ostrava, Ostrava, Czech Republic; 3 Department of Bacteriology and Mycology, Public Health Institute in Ostrava, Ostrava, Czech Republic; 4 Department of Anaesthesiology, Resuscitation and Intensive Care Medicine, University Hospital Ostrava, Ostrava, Czech Republic; 5 Department of Anaesthesiology, Resuscitation and Intensive Care Medicine, Faculty of Medicine, University of Ostrava, Ostrava, Czech Republic; 6 Department of Physiology and Pathophysiology, Faculty of Medicine, University of Ostrava, Ostrava, Czech Republic; 7 Department of Microbiology, Faculty of Medicine and Dentistry, Palacky University Olomouc, Olomouc, Czech Republic

**Keywords:** Invasive candidiasis, Molecular diagnostics, Biomarkers, T2Candida, β-D-glucan

## Abstract

Invasive candidiasis is a serious infectious disease that affects 2–10% of patients in the intensive care unit. It is caused by fungi of the genus *Candida*, and its diagnosis relies on multiple complex laboratory methods. Blood culture remains the gold standard for yeast detection in the bloodstream but has limited clinical utility because of its low sensitivity and prolonged turnaround time. Newer diagnostic approaches, such as molecular methods for *Candida spp.* DNA detection, which enable faster identificationbut still entail limitations, including variability in sensitivity. Another valuable tool is the detection of (1,3)-β-D-glucan, a polysaccharide in yeast cell walls; however, this marker lacks specificity. Timely initiation of appropriate antifungal therapy is crucial because delayed treatment is associated with increased mortality. Preventive strategies, including strict hygiene protocols and antifungal stewardship programs, are vital to reducing the incidence of invasive candidiasis. Emerging research on siderophores as candidate biomarkers for fungal infections indicates promising diagnostic potential. Given the complex pathogenesis and diverse clinical manifestations of invasive candidiasis, multifaceted diagnostic and therapeutic approaches are required. A combination of novel biomarkers, rapid molecular diagnostics, and optimized treatment strategies is essential to improve patient outcomes and reduce the complications associated with this life-threatening infection.

## Background

Invasive candidiasis (IC) is a serious infectious disease caused by fungi of the *Candida* genus, most commonly *Candida albicans*. Other medically important species include *C. glabrata (Nakaseomyces glabratus)*, *C. parapsilosis*, *C. tropicalis*, and *C. krusei* (*Pichia kudriavzevii*) [1]. In healthy individuals, these yeasts are typically present as commensal organisms on mucous membranes and in the gastrointestinal tract.

A series of pivotal steps underlie the pathogenesis of IC and systemic dissemination of infection. Two key steps are transformation of the fungus into a filamentous form, enabling the hyphae to penetrate host tissues, and the ability of *Candida* to adhere to surfaces such as epithelial cells or intravenous catheters and form a biofilm. Biofilms are complex structures that protect yeast cells against the host immune system and antifungal drugs, thereby inhibiting the effectiveness of these drugs. In IC pathogenesis, neutrophils and macrophages can effectively eliminate *Candida*, but in immunocompromised patients, immune system failure can lead to uncontrolled spread of infection. With candidemia resulting from contamination of intravenous catheters or indirectly through damage to the gastrointestinal mucosa, the infection has the potential to disseminate, leading to multi-organ involvement [2]. Several risk factors are associated with the development of IC, including immunosuppression with chemotherapy, organ or bone marrow transplantation, pancreatitis and abdominal surgery. Intravenous catheters, parenteral nutrition, invasive mechanical ventilation and broad-spectrum antibiotics, in conjunction with prolonged hospitalization in the intensive care unit (ICU), carry further risk. Specific conditions that increase risk include diabetes mellitus, chronic kidney disease, and liver cirrhosis [3]. Table 1 details the clinical manifestations of IC.

**Table 1:** Clinical manifestations of invasive candidiasis

Condition	Clinical signs
Candidemia	Presence of *Candida spp.* in the blood Fever, chills, hypotension, multi-organ failure; additionally, signs of heart failure may manifest
Endocarditis	Infection of the heart valve Exceedingly rare and associated with a high mortality rate Presents with persistent fever, heart murmurs, and signs of heart failure
Peritonitis	In patients undergoing peritoneal dialysis Presents with abdominal discomfort, fever, and abdominal distension Spontaneous fungal peritonitis in patients with liver disease
Meningitis	In patients with compromised immune systems Presenting symptoms include fever, headache, and neck stiffness, as well as neurological disorders
Other organ infections	Infections of the liver, spleen, kidneys, and eyes

Reference: Data adapted form [2]

Candidemia represents a significant infection of the bloodstream [2] and is the most prevalent invasive fungal infection in hospital settings, affecting both men and women at similar rates. A study conducted by the European Confederation of Medical Mycology of collated data from 64 hospitals across 20 European countries showed that the overall 90-day crude mortality rate from candidemia was 43% [4]. Furthermore, in that study, candidemia caused by rare *Candida* species, including *C. tropicalis*, *C. kefyr*, and *C. auris (Candidozyma auris)*, emerged as a potentially significant predictor of mortality in addition to established factors such as age and ICU admission.

Deep-seated candidiasis affects normally sterile areas of the body, such as the abdominal cavity or, less commonly, the chest cavity, and may or may not be associated with secondary candidemia [5]. Although pulmonary infections due to *Candida* are rare, involvement of the pleural space or mediastinum is recognized and generally classified within the spectrum of invasive candidiasis without positive blood culture [6]. Deep-seated candidiasis presents as a systemic inflammatory response, ranging from mild fever to septic shock with multi-organ failure. The clinical presentation depends on the affected organ(s). Diagnosis is based on the isolation of yeast from sterile sites, such as peritoneal or pleural fluid. However, the sensitivity of culture is limited, necessitating additional diagnostic approaches such as biopsy and histopathological examination.

IC remains a significant challenge in healthcare settings, particularly in immunocompromised patients and in those admitted to the ICU. Preventative measures are of the utmost importance to avoid infection and spread of resistant *Candida* strains within the ICU environment [7]. Key measures include adherence to antifungal stewardship (i.e., using appropriate antifungal treatment), which can limit the development of resistant *Candida* strains [8]. The identification and monitoring of *Candida* colonization are crucial for both infection diagnosis from sterile sites and colonization screening, especially in the case of resistant species such as *C. auris (Candidozyma auris)*. Regular testing for *Candida* in both sterile and non-sterile samples enables timely detection of epidemiological changes, shifts in species distribution, and the emergence of antifungal resistance. It is also advisable to perform antifungal susceptibility testing, including for isolates from non-sterile sites, to monitor potential resistance development [9]. Hygiene standards should include regular cleaning of equipment and ICU environments, the use of disposable materials, and strict adherence to hygiene procedures to help prevent the spread of resistant *Candida* strains. Isolation of patients colonized or infected with resistant *Candida auris (Candidozyma auris)* strains is essential to prevent transmission to other patients.

The pathogenesis of this infection involves complex mechanisms that enable *Candida* species to colonize, invade, and disseminate within the host. Understanding these processes, along with recognizing and mitigating the risk factors, is crucial for effective management and prevention. As the medical community continues to confront challenges associated with *Candida* infection, ongoing research and rigorous clinical practices are essential to improve patient outcomes and mitigate the burden of IC in healthcare settings, and particularly to enhance early diagnosis of IC.

This narrative review provides a focused summary of current diagnostic approaches for IC, particularly emphasizing molecular methods and selected biomarkers relevant to critically ill patients. The article does not follow a formal systematic review protocol but aims to highlight key themes and challenges in the evolving landscape of IC diagnostics.

## Diagnosis of Invasive Candidiasis

**Fig. 1:**
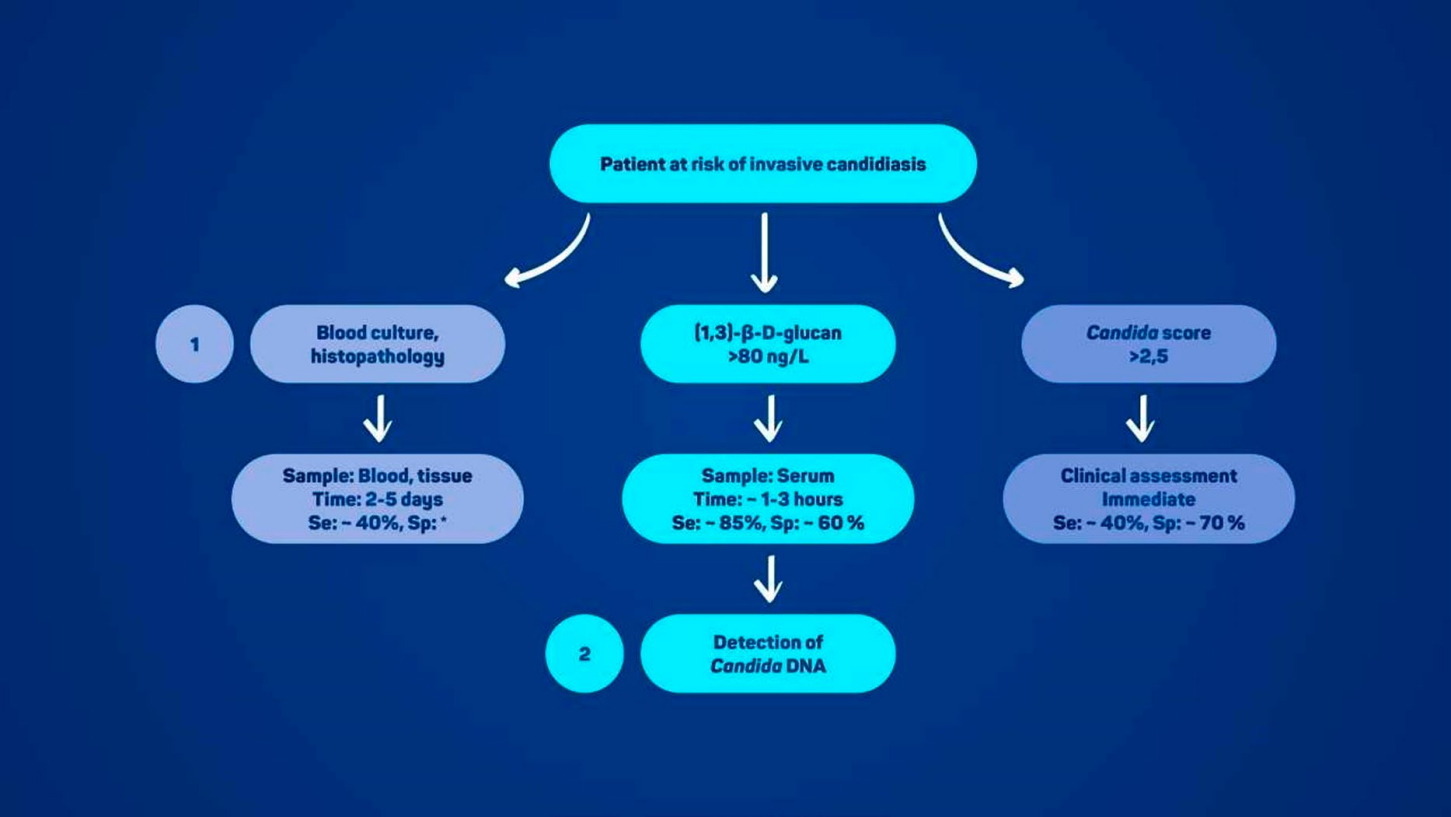
Schematic representation of the diagnostic algorithm for invasive candidiasis. The individual methods (clinical assessment, *Candida* score, culture and histopathology, (1,3)-β-D-glucan assay, detection of *Candida* DNA) are shown with information on sample type, approximate time to result, and estimated sensitivity and specificity. *Note:* The asterisk indicates the interpretative issue of blood culture sensitivity in invasive candidiasis without candidemia. The low sensitivity of blood cultures is the reason why the incidence of invasive candidiasis without a positive blood culture is estimated to be approximately 150% higher than that of candidemia. Moreover, there is no true gold standard for the diagnosis of invasive candidiasis with negative blood cultures, except for complete autopsy findings [6]

The diagnosis of IC represents a significant medical challenge, particularly in patients with sepsis, systemic inflammatory response syndrome, or severe immunosuppression, where rapid and accurate recognition of the infection is of paramount importance. The limitations of traditional laboratory diagnostic methods have led to the development of advanced techniques and new biomarkers for faster, more reliable detection. Delayed diagnosis and treatment are associated with an increased mortality rate [10], and the importance of early diagnosis and treatment initiation cannot be overstated. Figure 1 illustrates the diagnostic algorithm for using the various laboratory techniques in patients at risk of invasive candidiasis.

## Proof of Infection

### Gold Standard Methods for Diagnosing Invasive Candidiasis

The gold standard for diagnosing candidemia is blood culture in an automated system based on the detection of metabolic products and the multiplication of yeasts in blood culture vials. The quantity of yeast present in the bloodstream ranges from 1 to 10 colony-forming units per millilitre. Therefore, the yeast must be multiplied to yield a pure culture of a specific *Candida* species for precise strain identification and determination of its susceptibility to antifungal drugs. A limitation of this method is its low sensitivity, with a positive result observed in only 50–75% of candidemia cases [11]. Additionally, *Candida* requires 2–5 days to multiply, resulting in a significant delay in targeted treatment. In cases where antifungal therapies are used before blood collection, the findings are often false negatives.

Histopathological examination of tissues allows for the confirmation of IC and is highly specific, as the presence of hyphae in the tissues confirms the diagnosis. The principal disadvantage is that the method is invasive, requiring biopsy of tissues from otherwise sterile sites [2]. As yeast cells are unevenly distributed in tissue, low sensitivity is a disadvantage of this method, and the choice of sampling site affects the findings. This test is contraindicated in patients with thrombocytopenia and coagulopathy [12].

In contrast to these two gold standard methods for diagnosing IC, newer diagnostic approaches offer several advantages. Among these advantages are faster results with methods such as T2Candida® and PCR-based techniques, yielding identification within hours compared to the 2–5 days needed for blood culture. The T2Candida®, which was used in some previous studies, is no longer commercially available. Molecular methods and biomarker tests, such as (1,3)-β-D-glucan (BDG), often have higher sensitivity than blood cultures, especially when antifungal therapy has already been initiated. DNA-based methods can detect *Candida,* even when the organisms are no longer viable, which is not possible with culture-based techniques. Compared to histopathological examination, which requires tissue biopsy, blood-based biomarker tests or *Candida* DNA are much less invasive. Some molecular methods can identify multiple *Candida* species using a single test, and biomarkers, such as BDG, can provide early signs of invasive fungal infection before blood cultures become positive. Finally, some biomarkers may be useful for monitoring the effectiveness of antifungal therapy.

The newer methods also have limitations, including potential false positives, inability to determine antifungal susceptibility, and, in some cases, high costs. For these reasons, they are often used in combination with traditional methods rather than complete replacements.

### Candida DNA Detection as a Fundamental Methodology

Methods based on the detection of *Candida* DNA include a combination of DNA isolation from clinical material (whole blood or other body fluids) and subsequent amplification by PCR. This technology can identify yeast within hours, which is significantly faster than using blood culture. The disadvantages of this approach include the potential for false-positive results from contamination, inability to distinguish between live and dead organisms, and inability to determine susceptibility to antifungal agents [13]. In addition to in-house panfungal PCR assays [14], several commercial kits are currently available, including the Magicplex Sepsis Real-Time Test (Seegene), FungiPlex Candida (Bruker Daltonics), and SeptiFast LightCycler (Roche).

### T2Candida®

T2Candida® is a commercially available test (T2 Biosystems) for the rapid diagnosis of IC from whole blood K2EDTA, which has been approved by the U.S. Food and Drug Administration for clinical use. The diagnostic kit can identify the most common causative agents of IC, namely *Candida albicans*, *C. glabrata (Nakaseomyces glabratus)*, *C. tropicalis*, *C. parapsilosis*, and *C. krusei* (*Pichia kudriavzevii*). This method is based on the principle of T2 Magnetic Resonance (T2MR), which combines PCR amplification of yeast DNA with the subsequent binding of magnetic particles to the amplified DNA and signal detection by T2 magnetic resonance. T2Candida® has the advantages of speed and accuracy, but its disadvantages include the high acquisition cost of the T2MR instrument and the inability to determine sensitivity to antifungals [15]. Although T2Candida has shown promising results in the rapid diagnosis of invasive candidiasis, the authors acknowledge that the assay is no longer commercially available.

Zurl et al. reported a sensitivity of 68% for T2Candida® for candidemia [15]. However, in patients with a deep IC, the sensitivity was 7.7%. This significantly lower sensitivity has been corroborated by de Jesus et al. [16] and Clancy et al. [17], who reported respective sensitivities of 33% and 45% for the diagnosis of intra-abdominal candidiasis. As shown in Table 2, however, sensitivities vary widely across studies, ranging from 0 to 100%, depending on the patient population, definitions, and study design.

**Table 2:** Summary of studies evaluating the diagnostic performance of the T2Candida® and 1–3 BDG Fungitell® tests for the detection of invasive candidiasis in critically ill patients in intensive care units

Method	Sensitivity %	Specificity %	PPV %	NPV %	References
T2Candida®	80	97.1	62.5	98.8	Fisher et al. [19]
T2Candida®	73	96.4	NA	NA	Camp et al. [46]
T2Candida®	33.3	93.3	NA	NA	Lamoth et al. [47]
T2Candida®	42.8	94.2	75	80.4	Munoz et al. [48]
T2Candida®	100	100	NA	NA	Lucignano et al. [49]
T2Candida®	100	91.8	25	100	Giannella et al. [50]
T2Candida®	NA	NA	75	90	Helweg-Larsen et al. [51]
T2Candida®	NA	NA	50	96	Arendrup et al. [18]
T2Candida®	NA	NA	61	94	Krifors et. al. [52]
T2Candida®	0	85.7	0	96.8	de Jesus et al. [16]
1–3 BDG Fungitell®	94.6	75	87.5	88.2	Yazdanpanah et al. [53]
1–3 BDG Fungitell®	96.6	97.2	94.9	98.1	Dobiáš et al. [59]
1–3 BDG Fungitell®	9.1	85.4	3.0	95.0	Fisher et al. [19]
1–3 BDG Fungitell®	80	89.7	NA	NA	McKeating et al. [54]
1–3 BDG Fungitell®	89.7	75	78.8	87.5	Pini et al. [55]
1–3 BDG Fungitell®	83.3	89.6	62.5	96.3	Pazos et al. [56]
1–3 BDG Fungitell®	83.3	66.7	NA	NA	Lamoth et al. [47]
1–3 BDG Fungitell®	81.5	82.9	62.8	92.6	Fortún et al. [57]
1–3 BDG Fungitell®	64.4	92.4	83.8	75.1	Ostrosky-Zeichner et al. [58]
1–3 BDG Fungitell®	86	80	0.15	99.9	Eades et al. [20]

NA, not available; NPV, negative predictive value; PPV, positive predictive value

The specificity of T2Candida® for the diagnosis of IC exceeds 90% [18], indicating a low false-positive rate. This test also has a high positive predictive value of 62.5% and a negative predictive value of 98.8% for the detection of IC (Table 2) [19]. However, the definition of IC varies among studies, which may affect comparisons of sensitivity, specificity, and negative and positive predictive values of T2Candida®. The value of the test is maximized in patients with a high pre-test likelihood of IC [12, 17], and combination strategies are needed for the detection of the yeast cell wall antigen in the blood or other biomarkers of sepsis, such as acute-phase proteins or IC scores.

### Reliance of Current Methods on Yeast Cell Wall Polysaccharide Detection

The two most significant cell wall components of the *Candida* genus, mannan and BDG, are crucial polysaccharide biomarkers for IC diagnosis. They can be identified in patient sera by several commercially available assays.

#### Mannan

Mannan can be detected using an ELISA test (Platelia™ Candida Ag Plus, Bio-Rad Laboratories). Mannan is rapidly removed from the bloodstream and can form immunocomplexes with circulating antibodies to mannan, preventing detection of mannan antigen in serum or plasma samples. Thus, particularly in the early stages of IC, the sensitivity of these tests in patients at risk of IC is low, reported to be only 14% in a study by Eades et al. [20]. 

#### (1,3)-β-D-glucan

BDG is present in the cell walls of mycotic organisms other than yeast and thus is not an exclusive marker of *Candida*. Nevertheless, several studies have verified its sensitivity for diagnosing IC [16, 18, 20]. As summarized in Table 2, the reported sensitivities for BDG-based tests range widely from 9.1% to 96.6%, and specificities from approximately 65% to over 90%, depending on the study design, patient population, and applied cutoff values. Currently, the detection of BDG in serum or plasma is also recommended for the diagnosis of invasive fungal disease in adult patients in ICUs [21], where it is primarily employed to exclude IC (i.e., as a rule-out test) [22]. Two commercially available diagnostic kits are available for BDG detection, Fungitell® (Associates of Cape Cod, Inc.) and Wako β-Glucan Test (GT)® (Fujifilm Wako Pure Chemical Corp). The serum threshold value for diagnosing invasive fungal disease has been established at > 80 ng/L for Fungitell® [23] and > 7 ng/L for GT, respectively. It is recommended that BDG levels in patient serum be monitored over time.

A major limitation of BDG testing is the risk of false-positive results [24]. Elevated BDG levels may occur in the absence of fungal infection due to contamination from medical products containing glucans (e.g., gauze, surgical sponges, cellulose filters), administration of certain antibiotics (e.g., amoxicillin–clavulanate), or exposure to blood products and intravenous immunoglobulins. Hemodialysis using cellulose membranes, bacterial infections, and severe mucositis or translocation of gut flora can also contribute to false positivity. These factors considerably limit the specificity of BDG and necessitate cautious interpretation of results in the clinical context.

#### Candida Score

Although not a biomarker in the strict sense, the Candida score is a clinical prediction tool developed to help identify patients at high risk for invasive candidiasis in intensive care settings. The “Candida score” assists physicians in differentiating between *Candida* colonization and proven candidiasis in non-neutropenic ICU patients [25]. Scoring incorporates four factors identified as independent predictors of candidiasis. Each factor is assigned a specific value (coefficient) derived from logistic regression analysis, as follows: total parenteral nutrition, 0.908; surgery, 0.997; multifocal *Candida* colonization (*Candida* isolation from two or more non-contiguous foci), 1.112; and severe sepsis, 2.038. A Candida score > 2.5 indicates a high risk of established candidiasis, and the early administration of antifungal therapy is recommended. Patients with a Candida score > 2.5 are 7.75 times more likely to have proven candidiasis than patients with a score ≤ 2.5.

The scoring system was initially validated in a mixed population of ICU patients, of whom only 35% were admitted for internal reasons. As demonstrated by Laine et al. [26], the accuracy of this method may be compromised when applied to internal ICU patients. In their study, only 37% of patients with candidemia had a Candida score > 2.5, whereas 16% of patients with candidemia had a score of 0, 23% had a score of 1, and 24% had a score of 2. The most prevalent risk factors were severe sepsis/septic shock (53%) and multifocal *Candida* colonization (62%).

The Candida score is a valuable tool for the early identification of patients at risk for IC. For a comprehensive understanding of the patient’s risk profile, however, it is essential to recognize the limitations of this test and consider other risk factors, such as chronic liver disease and end-stage renal disease.

## Challenges and Future Perspectives in the Diagnosis of Invasive Candidiasis

In the last two decades, there have been significant advances in diagnostic methods for IC, but challenges remain in terms of sensitivity, specificity, and the need for rapid identification of the causative species. The development of standardized, clinically validated molecular-based tests is crucial for improving the diagnosis and treatment of IC, which could lead to better patient outcomes [12, 27–29]. The current literature emphasizes the need for rapid, sensitive, and specific diagnostic methods to improve patient outcomes in IC [21].

### Novel Detection Methods

In the past, the detection of secondary metabolites such as D-arabinitol appeared to be a promising avenue for diagnosing IC, offering potential improvements over traditional culture-based methods [30]. Although iron chelators have not been mentioned as diagnostic tools in this context, the importance of iron metabolism in *Candida* pathogenicity suggests that further research in this area could be beneficial [31–33].

Siderophores are small molecules with pivotal roles in iron metabolism in a range of microorganisms, including pathogenic fungi. These molecules can form strong complexes with iron, a vital component of fungal growth and survival. Consequently, siderophores are regarded as potential biomarkers for the diagnosis of invasive mycotic diseases [34]. The replacement of iron in siderophores with radionuclides, such as gallium-68, enables the targeted imaging of infections using positron emission tomography. With this approach, modified siderophores, including [68 Ga]Ga-TAFC and pyoverdin ([68 Ga]Ga-PVD), facilitate precise imaging of fungal and bacterial infections. Moreover, siderophores can be modified by the addition of fluorescent dyes for hybrid imaging or by conjugation with antifungal agents for targeted therapy.

A remaining challenge is to identify a unique siderophore for the genus *Candida* that can be used in diagnosing IC. In the quest for new *Candida spp.*–specific biomarkers, an untargeted CycloBranch-based approach should be used to allocate iron, zinc, manganese, and copper-containing molecules in bodily fluids [35]. The integration of these novel diagnostic tools with conventional methodologies may offer a more comprehensive approach to the diagnosis of IC, potentially ensuring the critical time necessary for establishing appropriate and timely antifungal therapy.

### Reducing Time to Diagnosis

A significant challenge for microbiology laboratories is to minimize the time elapsed between clinical sample collection and laboratory processing. Most microbiology laboratories do not operate on a 24/7 basis, and the typical hours of operation are from 7:00 to 16:00. Consequently, samples collected outside of these hours may be subjected to a processing delay of up to 15 h. For example, only 12% of microbiology laboratories in Europe operate 24 h a day to ensure continuous reporting of blood culture results [36]. A delay between sample collection and processing can affect the accuracy and speed of diagnosis, which is particularly important when rapid decisions are required, such as with IC. Blood culture results are essential for diagnosing candidemia and sepsis, and any delay may compromise timely initiation of treatment [37]. Uninterrupted operation of clinical microbiology laboratories is crucial in the context of technological advances in diagnosing IC and other infectious diseases. Early identification of the microorganism and determination of its susceptibility to antifungal agents enables physicians to prescribe targeted therapy, which reduces patient morbidity and mortality. Several studies have shown that most positive blood cultures are identified outside of the normal working hours of clinical microbiology laboratories [38, 39]. Despite the additional costs associated with employing more personnel and using advanced technology, the continuous operation of a clinical microbiology laboratory offers significant economic benefits. These benefits include a reduced length of hospital stay, decreased costs of ineffective therapy, and reduced risk of resistant yeasts and bacteria [40].

### Measuring Quality of Care

The EQUAL Candida score is a tool developed by the European Confederation of Medical Mycology to assist physicians in adhering to guidelines for the diagnosis and treatment of candidemia [41]. The EQUAL Candida score quantifies adherence to recommendations from the European Society for Clinical Microbiology and Infectious Diseases [42] and the Infectious Diseases Society of America guidelines [43]. The score incorporates the factors recommended for optimal treatment of candidemia. An initial blood culture (40 mL) should be conducted to identify the *Candida* species and determine antifungal susceptibility. Additionally, echocardiography, ophthalmoscopy, and treatment with echinocandin should be considered, with fluconazole administered if indicated by the antifungal susceptibility results. The duration of treatment is 14 days after the first negative follow-up culture, and the central venous catheter (CVC) should be removed if present. Finally, follow-up blood culture should be conducted daily until a negative result is obtained.

The EQUAL Candida Score is a system for assigning points for each item listed, with the overall score indicating the extent of concordance with the recommendations. The maximum score is 22 points for patients with a CVC and 19 points for those without a CVC. A higher score indicates greater concordance to the established guidelines. Huang et al. [44] found that a higher EQUAL Candida score was associated with superior concordance to recommendations and significantly correlated with patient survival. The mean EQUAL Candida score in their study was 8.91, which was significantly lower than the ideal value of 22. Patients who survived candidemia had significantly higher EQUAL Candida scores than those who died of infection. Furthermore, a cut-off EQUAL Candida score of < 10 was identified as a predictor of 30-day mortality, particularly in patients with CVC.

In their study, Zakhem et al. observed that CVC patients who survived had statistically significantly higher EQUAL Candida scores than those who died (13.1 ± 3.19 vs. 11.3 ± 4.77, *p* = 0.025) [45]. In patients without CVC, no statistically significant difference was observed in EQUAL Candida scores between survivors and those who died. Overall, the EQUAL Candida score was not a significant predictor of mortality in the patient population in that study. Further studies are required to assess concordance with recommendations for the care of patients with candidemia, and to evaluate the benefit of the EQUAL Candida score as an indicator for optimizing the care of patients with candidemia in hospitals.

## Conclusion

The diagnosis and management of IC remain a significant challenge in healthcare settings. The complex pathogenesis of this infection, coupled with the limitations of traditional diagnostic methods, underscores the need for advanced, rapid, and accurate diagnostic techniques. There has been substantial progress in the development of new diagnostic tools, such as T2Candida®is no longer commercially available, other PCR-based methods, and biomarker tests, each of which has its own advantages and limitations. Integration of these novel approaches with conventional methods offers a comprehensive diagnostic strategy that can potentially improve patient outcomes. The investigation of new biomarkers, including iron chelators and siderophores, presents promising avenues for future research and clinical application. These biomarkers could provide more specific and sensitive diagnostic tools for IC, particularly for differentiating between colonization and invasive infections. Furthermore, the importance of a timely diagnosis cannot be overstated. The continuous operation of clinical microbiology laboratories, although challenging to implement, could significantly reduce delays in diagnosis and treatment initiation. This approach, in conjunction with the use of rapid diagnostic techniques, has the potential to improve patient outcomes and reduce the healthcare costs associated with invasive candidiasis.

## Data Availability

No datasets were generated or analysed during the current study.
